# Vasectomy as a proxy: extrapolating health system lessons to male circumcision as an HIV prevention strategy in Papua New Guinea

**DOI:** 10.1186/1472-6963-12-299

**Published:** 2012-09-04

**Authors:** Anna Tynan, Andrew Vallely, Angela Kelly, Greg Law, John Millan, Peter Siba, John Kaldor, Peter S Hill

**Affiliations:** 1 Australian Centre for International & Tropical Health, School of Population Health, University of Queensland, Brisbane, Australia; 2 Public Health Interventions Research Group, Kirby Institute, University of New South Wales, Sydney, Australia; 3 Sexual & Reproductive Health Unit, Papua New Guinea Institute of Medical Research (PNG IMR), Goroka, Eastern Highlands Province (EHP), PNG; 4 International HIV Research Group, School of Public Health and Community Medicine, The University of New South Wales, Sydney, Australia; 5 Sexual Health and Disease Control Branch, National Department of Health, Port Moresby, Papua New Guinea

**Keywords:** Male circumcision, HIV/AIDS, Papua New Guinea, Health system strengthening, No-scalpel vasectomy

## Abstract

**Background:**

Male circumcision (MC) has been shown to reduce the risk of HIV acquisition among heterosexual men, with WHO recommending MC as an essential component of comprehensive HIV prevention programs in high prevalence settings since 2007. While Papua New Guinea (PNG) has a current prevalence of only 1%, the high rates of sexually transmissible diseases and the extensive, but unregulated, practice of penile cutting in PNG have led the National Department of Health (NDoH) to consider introducing a MC program. Given public interest in circumcision even without active promotion by the NDoH, examining the potential health systems implications for MC without raising unrealistic expectations presents a number of methodological issues. In this study we examined health systems lessons learned from a national no-scalpel vasectomy (NSV) program, and their implications for a future MC program in PNG.

**Methods:**

Fourteen in-depth interviews were conducted with frontline health workers and key government officials involved in NSV programs in PNG over a 3-week period in February and March 2011. Documentary, organizational and policy analysis of HIV and vasectomy services was conducted and triangulated with the interviews. All interviews were digitally recorded and later transcribed. Application of the WHO six building blocks of a health system was applied and further thematic analysis was conducted on the data with assistance from the analysis software MAXQDA.

**Results:**

Obstacles in funding pathways, inconsistent support by government departments, difficulties with staff retention and erratic delivery of training programs have resulted in mixed success of the national NSV program.

**Conclusions:**

In an already vulnerable health system significant investment in training, resources and negotiation of clinical space will be required for an effective MC program. Focused leadership and open communication between provincial and national government, NGOs and community is necessary to assist in service sustainability. Ensuring clear policy and guidance across the entire sexual and reproductive health sector will provide opportunities to strengthen key areas of the health system.

## Background

Large-scale clinical trials in Africa have shown that male circumcision (MC) has a protective efficacy of around 60% in preventing HIV acquisition in men [1-4]. The consistency and convincing nature of these findings, which confirmed earlier observational and ecological studies [5], has led to a rapid acceptance of MC within the international public health community [6]. In March 2007, the World Health Organisation (WHO) and the Joint United Nations Program on HIV/AIDS (UNAIDS) issued a formal statement of support for adult MC to be considered part of comprehensive HIV prevention strategies in high-burden settings [7].

Despite the clarity of this recommendation, the complex social and cultural context of Papua New Guinea (PNG) and the distinctive features of the epidemic and local responses to it mean that the lessons of Africa cannot be directly transposed to PNG where adult medical male circumcision (MC) is currently being considered by the National Department of Health (NDoH) as part of a comprehensive HIV prevention strategy. PNG has among the highest HIV prevalences in the Asia-Pacific Region, estimated to be around 1.0% among adults aged 15 – 49 yrs [8-15]. The epidemic in PNG is primarily linked to heterosexual transmission and exhibits substantial geographic heterogeneity, with over half the reported HIV diagnoses coming from Port Moresby, National Capital District; 20% from Western Highlands Province; and 10% from Morobe Province [14]. The development of effective and culturally appropriate strategies for HIV prevention is imperative given the geographical, linguistic and cultural diversity of PNG [9,16,17].

Evidence from previous acceptability studies for MC in PNG suggests that various forms of penile foreskin cutting in communities exist but that these do not commonly involve circumferential cutting and the excision of the foreskin [18-22]. Even in the absence of any formal health promotion, there is growing popular awareness of links between ‘circumcision’ and the prevention of HIV and Sexually Transmissible Infections (STIs) [20,23]. Health workers are already participating in a range of penile cutting practices, both formally as part of their public sector responsibilities, where they may perform procedures themselves, or manage the complications of procedures undertaken by others, and informally, as participants in community cultural practices. [21,23]. Given the complexity of this situation, and the already blurred boundaries between health workers, MC and other penile cutting, exploring the feasibility of scaling up existing MC activities without raising unrealistic expectations is deeply problematic. This research seeks to explore the experience of a comparable national program and review potential health systems lessons that could effectively inform the establishment of a national MC program.

### Vasectomy as a proxy

The already established PNG no-scalpel vasectomy (NSV) program, which started in 1997, provides a suitable model for comparison [24]. Vasectomy is a surgical method used to cut or tie the male vas deferens, resulting in permanent birth control [25]. Unlike the conventional technique, where one or two incisions are required, the no-scalpel technique involves skin puncture with a sharp-pointed, forceps-like instrument, with no sutures necessary to close the entry site [26]. This reduces adverse events, especially bleeding, bruising, hematoma, infection and pain and shortens the operating time [25].

The analysis of the NSV program offers a number of advantages within the complex health system of PNG:

· It deals with an established program, rather than projections based on assumptions.

· It allows for review of an existing program that addresses the relative technical difficulty of a surgical intervention compared to other prevention programs.

· It provides an analysis removed from direct discussions about MC and other HIV/AIDS initiatives in PNG which avoids raising expectations among health workers and communities that a MC program is about to be rolled out.

· It presents evidence for a retrospective analysis of issues around training, resource availability, financing, health workforce, and service delivery, in an analogous context.

By drawing attention to the broad range of influences on the health system demonstrated through the analysis of the NSV program, this study assists in providing a proxy framework on how a future MC program could exist within an already vulnerable health system.

In this paper we present the findings of a qualitative study conducted to explore lessons from the development and implementation of the NSV program in PNG to understand health system readiness for a potential MC program if it is deemed appropriate. This study is part of a multi-disciplinary, community-based research program to investigate the acceptability, epidemiological impact, cost-effectiveness and options for program implementation of MC for HIV prevention in PNG: the Male Circumcision Acceptability and Impact Study (MCAIS) which is being conducted by the PNG Institute of Medical Research in collaboration with the University of Queensland and the University of New South Wales in Australia.

## Methods

### Study design

**Figure 1:**
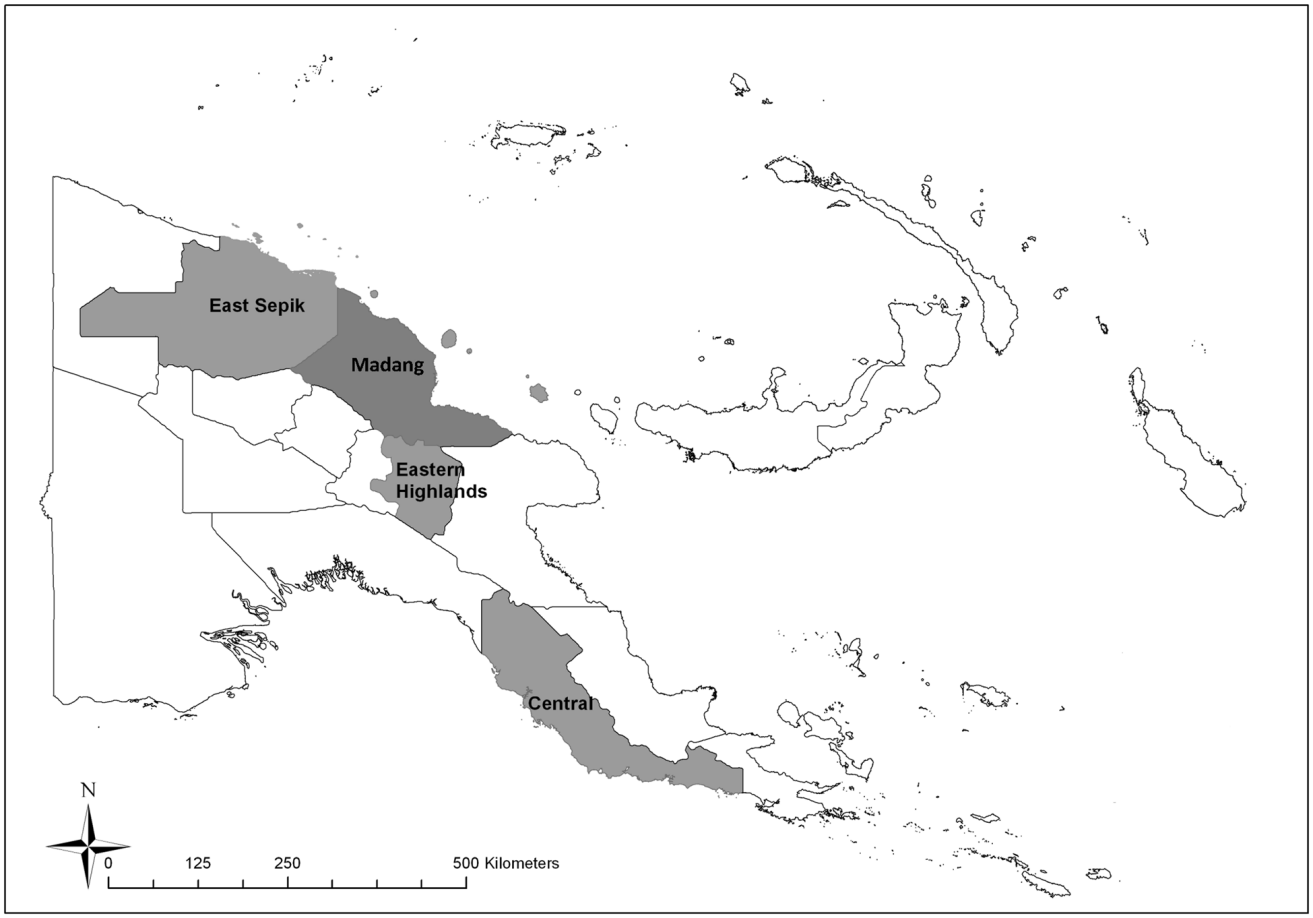
Map of Papua New Guinea with case study provinces highlighted.

Using a mixed method qualitative approach, data was collected over a 3 week period in February and March 2011 and combined a triangulation of qualitative methods including key informant interviews (KII); documentary and policy analysis; and structured and unstructured observations of identified NSV services. An iterative, purposive sampling technique was used to identify potential participants for the KII with upper level health system officials involved in the coordination of the national NSV program. Four NSV services were chosen as case studies based on reports of active service from the upper health system officials and included 1 non-government organization (NGO), 1 hospital clinic and 2 government outpatient clinics located in four provinces of PNG including, East Sepik Province: ESP, Eastern Highlands Province: EHP, Madang Province: MDGP and National Capital District: NCD (Figure 1). Following identification of the most active NSV services snowball sampling of frontline health workers (HWs) involved in direct implementation of the selected NSV services was completed.

The interview guide for the KII with upper health system officials followed a number of research themes including options for models of service delivery; human resource capacity, training and accreditation processes; supply and demand; strengths and weaknesses of the national program and funding arrangements. A separate interview guide was developed for the KII with frontline health staff and included further in-depth questioning of how the service operated; experience of being involved in the NSV service; perception of demand of service; and barriers and opportunities for implementing the program. The interview guides were developed in collaboration with AT, PH, AV, AK, JM and GL and revised on an ongoing basis to elicit more focused responses from participants and to accommodate themes that emerge in the early stages of data analysis.

All KII were conducted in English by AT and digital recordings of the interviews were taken and later transcribed. Two interviews were conducted over the phone to allow contact with key people who would have otherwise been difficult to access due to distance, responsibilities and time constraints. Documentary analysis included the review of raw data from the NSV services, training manuals, government reports, and national and provincial policies surrounding NSV and has been included in the results section. A field journal was kept during the intensive period of data collection to further ensure accuracy of findings and to record additional observations. Preliminary results were discussed with key members of the NDoH by the authors to ensure trustworthiness of results as the fieldwork progressed.

### Data analysis

The WHO framework of ‘6 building blocks’ of a health system [27], was used in the data analysis process to describe the experience of the NSV program in PNG and highlight the challenges faced. The building block framework was designed by WHO to promote a common understanding of what a health system is and constitutes the key components necessary for health systems strengthening[28]. The building blocks include service delivery; health workforce; health information systems; access to medicines, vaccines and technologies; financing and; leadership and governance [27]. In an iterative process the researchers organised the data into each WHO building block to review how the functioning of the NSV program in the country was impacted on by contextual factors. AT subjected the data to further thematic analysis with the assistance of analysis software MAXQDA (VERBI software GmBH, Germany) to organise data into identifiable themes within each of the 6 building blocks. The themes were discussed with all authors and adjusted where appropriate.

### Ethical implications

Ethical approval was granted by the Medical Research Advisory Committee (MRAC) in PNG and the Human Research Ethics Committees of the University of New South Wales and the University of Queensland. Written informed consent was sought from all individuals participating in interviews. Confidentiality was maintained in the data transcription process, which used pseudonyms to identify respondents. Consideration was given by all researchers to the customs, practices and legal systems in PNG, potential for language barriers and the provision of a clear understanding of research objectives to all participants involved in this study.

## Results

With the support of United Nations Population Fund (UNFPA), NSV has been operational in PNG since 1997 following the training of 25 PNG health care professionals in Indonesia to obtain an international certificate of NSV from the Association for Voluntary Surgical Contraception International (AVSC). The NSV program has been integrated within public health services and non-government organizations (NGOs), similar services that may be called upon to incorporate a MC program. However, the momentum to sustain a NSV program has been very difficult in PNG according to the results of 14 KIIs (frontline HWs, n = 7; upper level health system officials, n = 7) representing four NSV services in PNG (Table 1). Difficulties with transportation, access, staffing, training, financing and resources were all described as barriers. The following results are presented within the WHO building blocks of a health system and include the analysis of key documents including government reports and policy documents.

**Table 1:** Overview of four NSV services that took part in this research

**NSV Service**	**Program Design**	**Service Leadership**	**Frontline Workforce**	**Service Delivery**	**Promotion of Service**	**Estimated Annual Service**	**Finance**
**Eastern Highlands Province (Government Service)**	Stand alone service situated within family health services program	Provincial health	2 x CHW	· Clinic 2 x per week	· Peers/ Word of mouth	~400 per year	UNFPA
		Family health services coordinator	1 x NO	· Outreach when requested	· District CHW		HSIP
**Central Province (NGO service)**	Aligned with men’s sexual and reproductive health program	International NGO	1 x CHW	· Rural outreach service.	· Men’s health clinic.	~ 2 per year at urban clinic	NGO supported
			3 x NO	· Saturday clinic when available	· Promotion during outreach	~ 100 per year in rural areas	Small fee for service
			1 x visiting medical doctor		· Peers/word of mouth		
					· District CHWs		
					· Incentives for health worker and clients		
**Central Province (Urban Government Hospital)**	Aligned with Obstetrics and Gynaecology Department	Hospital Department Head	Medical doctors	· As requested and when available	· Wives/mothers	~ 40 per year	Government
					· Peers/word of mouth		
**Madang Province (Government Service)**	Stand alone service situated within family health services program	Provincial Government	2 x CHWs	· No set clinic	· Peers/word of mouth	Between 200 and 300 a year	NGO Support for training and outreach
		Family Health services coordinator		· Outreach as required	· Promotion during outreach		HSIP
					· District CHWs		UNFPA
		Technical assistance from International NGO			· Incentives for clients		

NO: Nursing Officer; CHW: Community Health Worker; HSIP: Health Sector Improvement Program.

### Leadership and governance

No-scalpel vasectomy (NSV) provides an important component of the NDoH family planning program for safe motherhood, as well as a strategy to address population growth in PNG (an identified concern since independence in 1975). The NSV program is overseen by the NDoH with the decentralized health system resulting in provincial governments being responsible for administrating and prioritizing the service at a local level. NSV has been highlighted within numerous PNG policy documents including the National Health Plan (2011–2020); Reproductive Health Policy (2009); National Family Planning Policy (2007); and National Population Policy (2000–2010); to assist in reducing the burden of maternal mortality and morbidity in PNG, as part of the Millennium Development Goal to improve maternal health. However despite this support, the NSV program competes with a number of other priorities within provincial and national health departments. As a result, control of the program as a nationwide effort is often underpinned by provincial and district level priorities resulting in mixed results throughout the country. Table 1 describes the structure and leadership of the 4 NSV services analysed.

National level officials acknowledged that there were historical difficulties for effective governance and leadership of the NSV program for various reasons. In particular, leadership on strengthening the health system in general was seen as a much needed priority for the success of any program.

"Getting the health systems right in the first place is a priority. Because at the moment our health systems are in tatters, they have collapse, anything outside a couple of successful provincial hospitals. And nothing else much works."

"KII upper health system"

Other key issues highlighted by upper lever health system officials include the effectiveness and efficiency of the government to organise an effective program, including promotion and implementation Table 2 (quote 1 & 2). Being able to translate policy into practice was also acknowledged by one government official as a key issue.

**Table 2:** Illustrative quotes for Leadership, Governance and Health Financing

**Quote 1**	Well it [The NSV Program] has never been organised.... it has just never been organised properly. We have tried to make inputs on so many occasions for so many years. But family health services have just never been competently organised themselves.
	**KII Upper Health System Official**
**Quote 2**	I think the other challenge is awareness, people understanding of the importance of family planning and the issues surrounding the permanent methods of family planning. So those are challenges that we face and specifically on no-scalpel vasectomy. Since 1997 we have had a number of our staff were sent to Indonesia to be trained and now there are only a few you know that are taking the initiative forward. But the rest have gone over and basically because of the difficulties that they face in facilities that are not in a good state for them to perform, or they don’t get the support they need. There are a whole lot of other issues.
	**KII Upper Health System Official**
***Quote 3***	I mean I am sure that money could be accessed if we really put our minds to it. First of all you need to produce a national program. And then you need to get funding for it. Instead of having UNFPA passing by and saying would you like some funding to do a bit of vasectomy and the answer is always… yes please. And then, then nothing happens. They throw a bit of money at it and they do a bit of something. That is usually fairly ineffectual. Like they run a little vasectomy training program and the people in the program never get to do a vasectomy .... It’s just hopeless.
	**KII Upper Health System Official**
**Quote 4**	Yeah, about this funding we do normally get this funding from the health department, but we are finding it difficult. For me as a vasectomist, to go out within the district to perform a service to the men and at the same time perform hands on training to the apprentice I need continued funding. Funding is really a problem within this program and within the province.
	**KII Community Health Worker**
**Quote 5**	Sometimes they tell us that there is no money and we have to wait. Vasectomy, comes under the family health services. The program safe motherhood and so the vasectomy program comes under that safe motherhood family health services. I really don’t know how much we have, but we are just doing the proposal. And it’s up to the people up there, the finance team to approve our patrol [outreach service] and then they give us the funding.
	**KII Nursing Officer**

"Family planning has been identified as an important issue. So in terms of priorities it is in the policy document. Now it is the question of translating it into actual operations and this does rely on the capacity of the facilities. However, you also have got national provincial and district government. So we can come up with a policy or a statement saying that the priority is this, but when it goes to actual service delivery it depends if it is a priority at the other levels. There may be other challenges that they may face."

"KII Upper Health System Official"

Sustainability of services was often described to be heavily reliant on key drivers within provincial government and frontline health services as well as ongoing financial commitment from both provincial and national governments. As one provincial health official who worked within a province with a successful NSV program explained:

"At the provincial level, we have a budget for family health services because one of our priorities is family health services including safe motherhood. Vasectomy falls within this. Because now we can see that our population is increasing at a very alarming rate. And compared with economic development, the population is just growing miles. We understand this, so in this province one of our priorities is the family planning program."

"KII Provincial Health Official"

The impact of poor leadership was evident with many of the frontline health workforce. According to frontline HWs interviewed, feelings of poor support from government including difficulties accessing funds and resources was often a problem. As a result, many had difficulties with maintaining momentum for the service.

"…I could have done better but I got stranded, logistically, the transport and all this. And the thing is the province they didn’t give me enough support. And also in my district, even though they knew NSV was very important, they couldn’t fund it. They couldn’t support me."

"KII frontline HW"

### Health financing

Funding for the NSV service has been historically sourced from a variety of government and multinational organizations, in particular the UNFPA and Health Sector Improvement Program (HSIP). With funding coordination at national government level, provincial governments are responsible for obtaining money and allocating it to the service accordingly. Participants reported ongoing difficulties with accessing funds to support NSV activities including supervision of newly trained health workers, providing outreach services and obtaining equipment Table 2 (quote 3, 4 & 5). This was not only described as being due to actual availability of funds, but also to the limited maneuverability of funds through the system, resulting in reported delays:

"So we have no funding for the training and we are still waiting, I don’t know where the funding will come from. The funding will come, but from which source I don’t know. I am still waiting. I will just wait."

"KII frontline HW"

In two of the provinces reviewed, NGOs have been able to bridge the gap by providing financial or technical support to the training program or outreach services. Maries Stopes and Pathfinder International are the only two NGOs currently operating in PNG that have become involved with the NSV program. Pathfinder International provides financial support for training and allowances for outreach services in Madang Province. Marie Stopes operates at urban clinics and provides peri-urban outreach services in Port Morseby, NCD, as well as supporting NSV programs in more remote areas of Central Province. Small user fees were required by one NGO to access the service. NGO support is not available in all provinces but some provinces, such as EHP, have successfully implemented NSV programs without direct local-NGO support.

### Service delivery

**Figure 2:**
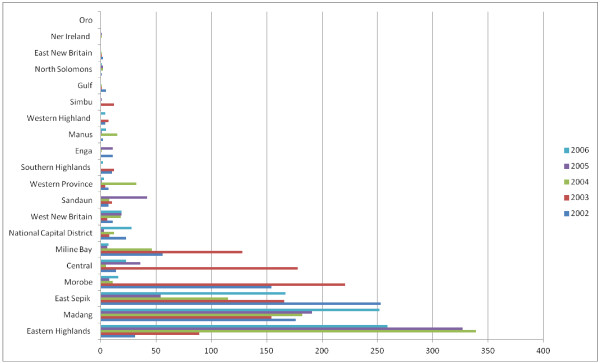
**No-Scalpel Vasectomies carried out in Papua New Guinea, 2002 – 2006.** [Adapted from O'Connor, M. (2007).].

Systemic issues such as funding, and provincial and national government support resulted in uneven service deliver across provinces and over time (Figure 2). Other key factors were poor supervisory support and training for staff; staff migration; inadequate program monitoring and evaluation; poor integration of the NSV program with other health services; and transport and logistical difficulties.

High demand and increasing waiting lists for the NSV service were commonly experienced in rural areas. However, waiting lists also existed in urban settings due to irregular financial support of designated staff, as well as inflexible times at which the service could be offered:

"Last year, we had two clients that were booked for vasectomies, but the doctor who was going to do the vasectomies wanted to do it on a Saturday, and these two guys, they go to church on Saturdays, so they couldn’t come. And they are still on the waiting list."

"KII frontline HW"

Raising expectations within communities for services through promotional activities resulted in a construction of obligation between worker and potential client. With increasing awareness and demand, but no service to support this, creation of dissatisfaction within the community became evident. For example, one respondent described an incident where he was confronted by a man who was particularly frustrated with waiting for the service.

"One client arrived here with a very bad temper. And he says “I am waiting and waiting and I have already four children.” He wanted to try and sue me for not arranging to go to him [in his village] because while he was waiting he had two sets of twins. And that made him really angry. So he came and said “your program is not a good program.” So he was not just talking about the procedure itself but the waiting that existed for us to go out and do the procedure. That was one of my struggles that I had."

"KII frontline HW"

According to frontline HWs an increasing demand for NSV services had been observed and was believed to stem from increasing economic pressures and demand for land experienced in communities as people continued to have large families. One HW also reported that after providing NSV services within a small community, he discovered that some men appeared to have been motivated by a desire to have more sexual partners without the fear of having more children.

### Health information systems

A challenge with developing efficient and effective health information systems in PNG was a concern for all upper health system officials.

"Our population is so scattered and so remote, we try to build up our surveillance as much as possible. And because of the remoteness of our communities it is a great challenge to us. And try to build up a system that will enable us to get information quickly, to respond to it quickly. But you know it has been a great challenge to us."

"Upper Health System Official"

This was evident in the review of the NSV program with details of national statistics for NSV services difficult to obtain prior to 2002. Estimates derived by triangulating data from several sources, suggest that the data recorded in Figure 2 may represent only about 50% of the total number of vasectomies conducted. This raises questions about demand:

"…the national picture for me at the moment is, well I know that the demand is there for vasectomy services and that there are limited service providers. That is the general picture, the general statement I can make. But in reality in terms of hard data, I cannot prove that."

"KII upper level health official"

Numbers on waiting lists were also often difficult to ascertain. No data on adverse events, including infection rates or failure rates, appeared to be systematically collected by any of the NSV services reviewed. Post-operative monitoring was also rarely completed due to the difficulties of access, population mobility and limited ability to contact people in PNG e.g. lack of mobile phone ownership or access in rural areas. These difficulties were noted to be a significant problem by all upper level health officials.

### Access to essential medicines, equipment and other resources

All frontline HWs reported that access to essential medicines and equipment for the NSV service was not a real problem despite upper level health officials suggesting that access difficulties were a common problem in PNG and likely had a significant impact on NSV service provision. However frontline HWs did report significant difficulties in accessing drugs for other components of the family planning service.

"I order the drugs through the local guys, but they told me that the won’t be ordering drugs for family planning commodities. Now family planning drugs are coming from Moresby. Like the vaccines. So I ordered them in November and I still haven’t got the supplies here [4 months later]. And ah we called this morning and they said the person who is in charge for doing the order for the, the family planning orders, he has been sacked or something. So now we are running out of things and have to send people elsewhere."

"KII Nurse"

Lack of transport for the NSV program, particularly for outreach visits, was considered more of an important obstacle by all frontline HWs. To deliver outreach programs, the HWs reported needing to rely on vehicles from other programs to assist.

"Equipment and drugs for the NSV I would not say is really bad, I think there is enough. But the biggest problem is that we do not have transport. At one stage, I thought that I was going to get a vehicle so I started planning my program to go from district to district there, and not be restrictive to the district here.However, it was given to someone else."

"KII CHW"

### Health workforce

Expanding human resources has been a key objective of government policy since the initiation of the NSV program in 1997. HWs at different levels have been trained including doctors, health extension officers (HEOs), nursing officers (NOs) and community health workers (CHWs). Difficulties with attrition of trainees and in implementing the NSV accreditation program continue to be reported by both frontline HWs and upper health system officials.

"I have trained health workers but we don’t know how they progress. I have no idea where they are now, or whether they are performing the procedures or not. So it is a sad story to me… It’s a waste of funds and all that, just training them and leaving them like that."

"KII frontline HW"

Training and support supervision are provided by a small number of clinicians in PNG and even fewer clinicians are currently able to certify HWs as vasectomists. A certified vasectomist would be expected to have demonstrated competent skills by completing 50 supervised vasectomies (fewer than 50 if they are a medical doctor) as well as proficient theoretical knowledge to implement the procedure. However, with limited supervisors available, many HWs who have received training have subsequently been left unsupervised and uncertified. Little information was available about what happens to those who are trained but unable to complete the supervision and certification program. This was a concern of both upper level health officials and frontline HWs.

"The training part is easy, we can do that. The difficult bit is the follow up. Follow up supervision. That has been really poor. Even from the beginning. I was in Madang, we trained 50 or 53 health workers. And so far we have only certified two of them."

"KII Upper level health official"

These difficulties with implementing the training and supervision program were reported as due to a lack of staff to deliver the program, but also to difficulties with accessing funds and general travel to areas in PNG. Frontline HWs who have been certified all describe long periods of waiting to be officially certified and recognized as a vasectomist.

"I am a CHW. I did my training on vasectomy as hands on training. And they certified me with a certificate from UNFPA; they gave me a certificate last year, early last year. But I went previously for more than 10 years, on vasectomies before that."

"KII frontline HW"

There were also reports from respondents admitting to performing unsupervised NSV, despite not being officially certified.

Upper level health officials acknowledged the importance of frontline HW’s initiative and dedication to their post-training program. The onus on frontline HW to promote services within the community and to maintain strong communication with trainers and upper level health officials, was considered an important factor in staff retention difficulties and an inability of the service to prevent staff migration to other programs.

It was unanimous among all upper level health officials and those involved in training that among all HWs, CHWs were the most likely to remain dedicated to providing and sustaining NSV services. This was considered a result of their work being conducted directly within communities.

"CHWs are interested, because they are right at the post where the population is. So it really acts like something that is very important to them, that is why they have been so successful."

"KII frontline HW"

Community engagement was identified as an integral part of a successful NSV program due to CHWs ability to promote the service.

## Discussion

This analysis of four active NSV services currently operating in PNG provided an opportunity to consider the health systems implications of a national MC program without raising expectations that such a program was about to be introduced. The results suggests caution in implementing a future MC program, given obstacles in funding pathways, inconsistent support by government departments, difficulties with staff retention and erratic delivery of training programs With no current national MC program in PNG, and previous research indicating penile cutting as part of the cultural landscape, the underlying complexities in health service delivery in PNG uncovered in the analysis of the NSV suggests that focusing on strengthening the health system was of paramount importance before attempting to initiate and roll-out any new prevention technologies.

### Health system readiness

According to mathematical modelling research, it is predicted that male circumcision will have a moderate impact on the PNG HIV epidemic overall with benefits reduced or eliminated by changes in sexual behavior [29]. Therefore, health system readiness and good leadership is integral to the success of aligning MC as part of a comprehensive HIV strategy. Difficulties with management of the NSV program were described and due in part to competing responsibilities of key upper lever health official coordinators and frontline staff which impacted on planning, training and obtaining funding. If MC is introduced for HIV prevention in PNG, ensuring understanding of the program at multiple levels of government, particularly at provincial level, will be essential to future program success. Identification of key workers in provincial areas with clear and strong support from the NDoH will assist in maintaining momentum and support.

Effectively mobilising human resources has been a widely discussed topic in the implantation of a MC program in African countries [30-32]. This study demonstrated that sustained NSV services relied significantly on the motivation and support of frontline health workers, in particular, CHWs, whose ability to gain community support, promote awareness and provide successful services should not be over looked. Task-shifting to increase human resource capacity has been identified as a potential option in a number of African countries attempting to implement MC programs and has relevance to PNG[33,34]. The introduction of MC for HIV prevention in PNG will require significant additional human resourcing and may benefit from considering how best to engage CHWs, who currently represent around 35% of frontline HW staff [35]. In an already vulnerable health system, significant investment in equipment and negotiation of clinical space also needs consideration, a similar finding in other capacity studies in Africa [30,32,36-38].

Of particular concern for a future MC program, poor monitoring and recording of men who have undergone NSV, as well as identification and follow up of trained HWs, were recognised as problems in the current NSV program. Unevenness of NSV service provision appeared to exist across provinces and over time, however, a clear picture of the national program was difficult to obtain due to gaps in recorded statistics. The need for systematic monitoring and evaluation of MC services across government and NGO sectors is important, to observe gaps in workforce and other resources; to determine if priority targets are being met; and to identify changing demands or other issues within the program [39].

The challenges of implementing national MC programs in Africa have also centred around the ability to access sustained domestic and international funding with leadership and visible champions at all levels to mobilise essential for maximum effect [31,32,40]. Given the existing popular market for penile cutting in PNG, a sustainable population-based MC service may justify the introduction of a nominal user fee with a small user fee charged by one NGO for the NSV an observed possibility. However, sustainable funding of a targeted MC program may have to consider alternatives that would not exclude vulnerable, disadvantaged clients because of cost.

### Strengthening relationships

The role of NGOs in the roll out of MC programs in Africa has not been fully explored; however the potential benefit of an integrated approach needs further consideration. The success of NGO efforts in technical and financial support to strengthen service delivery in key provinces in PNG for NSV may be applied to a national MC program. For example, dilemmas were reported around access to appropriate transport particularly for rural outreach visits and concerns were raised around access to enough medicines and essential equipment. Partly out-sourcing a future MC program to the NGO sector in PNG could provide an opportunity for technical and financial assistance in managing key issues identified in the NSV study.

In Kenya, consistent political support and ongoing community consultation have allowed implementation challenges for MC to be addressed as they arise [41]. The need to develop close relationships with communities is integral for program success across PNG and would need to be a focal point of implementing an invasive intervention such as MC. Promotion of NSV that was not followed up with an immediate service was shown to threaten community support for the program. Careful management of waiting lists and understanding the unique travel and access constraints present in much of PNG are required. Ensuring cooperation of services, particularly in urban centres, will assist in managing changes in demand, and corresponding waiting lists, as MC is rolled out.

Evidence of formal incentives for clients and HWs were observed and contributed to the promotion of the NSV service. However, difficulties with understanding motivations in clients were also reported by frontline HWs. As health workers were the only ones interviewed in this paper, minimal conclusive insight could be provided towards the community perceptions of NSV and the reasons for accessing the service. The impact of socio-cultural factors such as religion, traditional and contemporary understandings of family planning and the prevention of disease requires further exploration. The motivations for men accessing a MC program will need to be carefully monitored due to the potential for the program to result in reductions in condom usage, although such risk compensation has not been observed in other settings [41-45].

A dedicated men’s health service or clinic appeared to have the most success in promotion, access and uptake of NSV. With the minimum package for MC services as defined by WHO including voluntary counselling and testing (VCT); treatment and exclusion of STIs; and safe sex counselling (including provision and promotion of male and female condoms), a number of other opportunities arise for managing men’s sexual health in PNG [46]. Promoting MC through male clinics as part of family health services and sexual health services, or establishing culturally relevant programs in individual provinces such as through mediated “Haus Man” (traditional male initiation) projects may be valuable options for future service delivery.

### Limitations

The research project involved qualitative research methods. However, as with the nature of qualitative research, data results are limited in their ability to be generalized to the wider population. The case study analysis of the NSV program in PNG utilised by the researchers has advantages, but also some limitations. Studying a case poses challenges since it involves more variables than data points. The case study approach was also constrained by factors impacting case selection including a limited number of known NSV services identified by the NDoH; available time; and access to the services. However, the case summaries presented provide a general picture of the situation at a particular point in time and an opportunity to better understand casual factors that enable or hinder similar health programs.

## Conclusion

Analysis of the NSV program in PNG reveals a history of complexities around funding, resourcing, service delivery, management and sustainability of the national program. Lessons from this experience show that a number of health system priorities need to be addressed before a national MC program can be considered. Understanding how to effectively utilise and support staff in a sustainable way is integral to the success of the program. Decisions about service delivery models should acknowledge not only the cultural sensitivities of the communities but also the likely impact on health resources that a MC program would have on this already vulnerable health system. Defining responsibilities for sustainable funding, program leadership and the role of non-government and development partners will be essential. Ensuring clear policy and guidance across the entire sexual and reproductive health sector will provide opportunities to strengthen key areas of the health system.

## Competing interests

The authors declare that they have no competing interests.

## Authors’ contributions

PH, AT & AV participated in the conception of study design. Technical assistance was obtained from GL, AK and JM. Fieldwork was conducted by AT. Data analysis was carried out by AT with assistance from PH & AV. The manuscript was drafted and revised with support and contributions from PH, AV, AK, GL, JM, PS, & JK. All authors have read and approved the final manuscript.

## Pre-publication history

The pre-publication history for this paper can be accessed here:

http://www.biomedcentral.com/1472-6963/12/299/prepub
